# Association between reported aetiology of central nervous system infections and the speciality of study investigators—a bias compartmental syndrome?

**DOI:** 10.1093/trstmh/try008

**Published:** 2018-02-21

**Authors:** Tehmina Bharucha, Serena Vickers, Damien Ming, Sue J Lee, Audrey Dubot-Pérès, Xavier de Lamballerie, Paul N Newton

**Affiliations:** 1 Lao-Oxford-Mahosot Hospital-Wellcome Trust Research Unit (LOMWRU), Microbiology Laboratory, Mahosot Hospital, Vientiane, Lao PDR; 2 Division of Infection and Immunity, University College London, London, UK; 3 Section of Infectious Diseases and Immunity, Imperial College London, UK; 4 Centre for Tropical Medicine and Global Health, Nuffield Department of Clinical Medicine, University of Oxford, Churchill Hospital, Oxford, UK; 5 Mahidol-Oxford Tropical Medicine Research Unit, Mahidol University, Bangkok 10400, Thailand; 6 UMR ‘Unité des Virus Emergents’ (UVE: Aix-Marseille Univ–IRD 190–Inserm 1207–IHU Méditerranée Infection), Marseille, France

**Keywords:** Bias, Central nervous system infection, Encephalitis, Epidemiology, Meningitis

## Abstract

**Background:**

Conventional descriptions of central nervous system (CNS) infections are variably categorized into clinical syndromes for patient investigation, management and research. Aetiologies of the most commonly recognized syndromes, encephalitis and meningitis, tend to be attributed predominantly to viruses and bacteria, respectively.

**Methods:**

A systematic review was performed of aetiological studies of CNS syndromes and data extracted on reported author specialities.

**Results:**

The analysis identified an association between the author’s speciality and the CNS syndrome studied, with a tendency for virologists to study encephalitis and microbiologists to study meningitis.

**Conclusions:**

We suggest there is bias in study design. Stronger multidisciplinary collaboration in CNS infection research is needed.

## Background

Aetiologies of the common central nervous system (CNS) infection syndromes of encephalitis and meningitis have tended to be attributed predominantly to viruses and bacteria, respectively.^1–3^ In Laos, we have not found an association between syndromes and aetiologies and no significant difference in the proportions of detectable viral and bacterial aetiologies (Dubot-Pérès et al., submitted).

The significance of viral infections in the presentation of meningitis is becoming better characterized.^4–6^ Data suggest the detection of viruses in at least 50% of patients with clinical meningitis.^7^ Conversely, 20% of the 1570 encephalitis patients enrolled in the California Encephalitis Project were diagnosed with a bacterial infection.^1^

If virologists are not involved in diagnostic testing, a virus may not be identified. Indeed, it is notable that guidelines for the management of meningitis do not consistently feature virologists, nor do encephalitis guidelines involve microbiologists.^2,3^

We hypothesized that the reported aetiology of encephalitis and meningitis may be associated with the speciality of the study investigators. We performed a systematic literature review of studies examining the aetiology of encephalitis or meningitis and extracted data on the speciality of the investigators to investigate if this impression is correct.

## Methods

Searches were performed in PubMed, Embase and Cochrane, accessed using Ovid gateway, and references from relevant articles from January 2000 to April 2017 using the MESH and keyword search terms (‘encephalitis’, ‘meningitis’, ‘brain infection’ or ‘Central Nervous System infection’) and (‘epidemiology’, ‘cause’, ‘aetiology’ or ‘etiology’). Selection criteria included English-language epidemiological studies investigating the aetiology of community-acquired meningitis or encephalitis in human patients. Each paper was described as concerning meningitis or encephalitis depending on the presence of these keywords in the MESH and keyword search terms. If both terms were present the paper was excluded, as were case reports (defined as fewer than four patients), studies of meningoencephalitis, specific infectious agents or groups (e.g., case series of *Streptococcus suis*, HIV or viral meningitis) and brain abscesses. Two authors independently assessed studies for inclusion and extracted data on the speciality of all authors and a third author resolved disagreement. Searches were reported according to Preferred Reporting Items for Systematic Reviews and Meta-Analyses (PRISMA) guidelines.

The speciality of each author was derived from his or her departmental affiliation stated in the publication. Specialities were recorded as microbiology, virology, infectious diseases, neurology, paediatrics, research/public health and other. Bacteriology was not described as an affiliation in any paper and we assumed that microbiology did not include virology as a subspeciality. The data were summarized for each study as one point for a speciality if one or more authors reported an affiliation for that speciality. Results were analysed in Excel (Microsoft, Redmond, WA, USA) and Stata 14 (StataCorp, College Station, TX, USA) and presented as the number and percentage of the total included papers. Papers were further categorized as those whose investigators included ‘microbiology without author virology input’, ‘virology without author microbiology input’ and ‘other’. The association between these three categories and two clinical syndromes, encephalitis and meningitis, as defined in the paper were analysed using a Fischer’s exact test and reported with the corresponding p-value.

## Results

**Figure 1.**
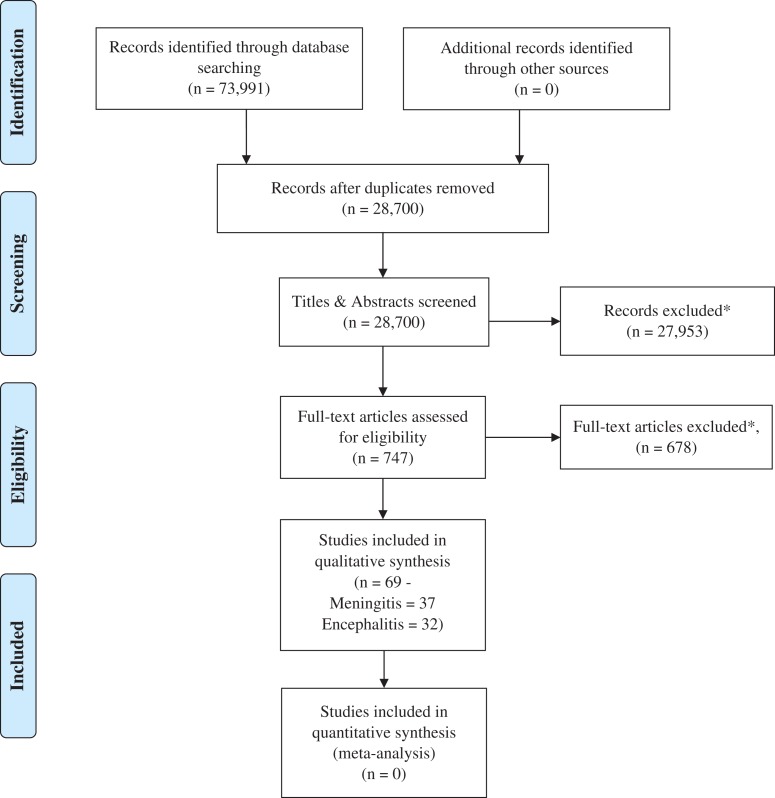
PRISMA flow diagram of the study selection for the systematic review. *See text for full exclusion criteria.

Sixty-nine studies were included in the analysis, 32 investigating encephalitis and 37 investigating meningitis (Figure 1).

The median number of authors was 5 (interquartile range 4–8) and the total number of individual authors from all the studies was 410. The studies were most commonly (29 [42%]) performed in the UK and USA, with the remainder performed in diverse countries (Table 1).

**Table 1.** Locations of the included studies. Light shading represents low- or low-middle income status and dark shading represents upper-middle or high-income status, as per the World Bank Definition 2018^9^

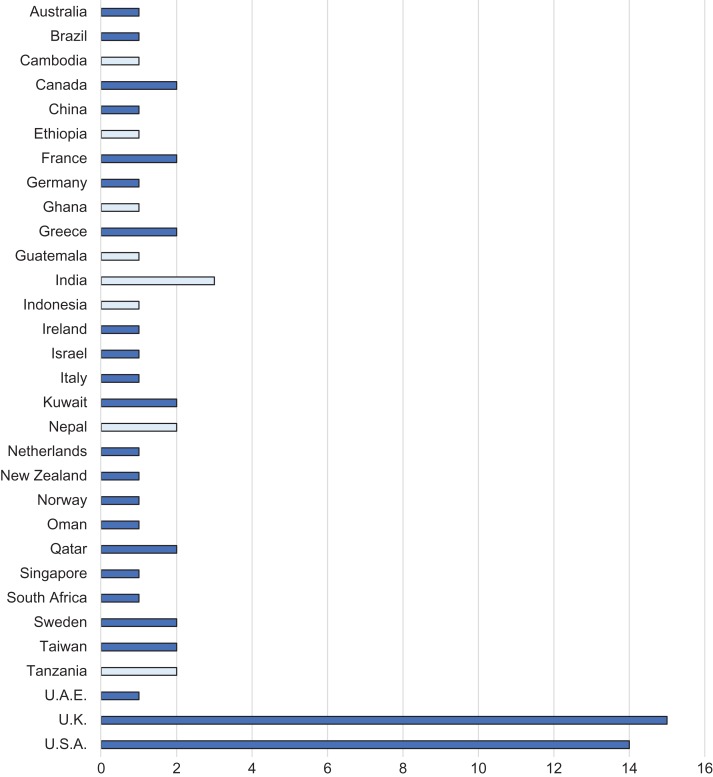

Seventeen (24.6%) studies received a contribution from a microbiologist author, 6 (8.7%) from a virologist and 16 (23.2%) from an infectious disease specialist (Table 2). There was also considerable involvement of neurologists, paediatricians and specialities such as public health and emergency medicine. However, 38 (55%) of the studies had no apparent microbiology, virology or infectious diseases author listed.

**Table 2.** Speciality of the study investigators included in the papers by clinical syndrome

	M		V		ID		N		P		R		O		Total	
	n	%	n	%	n	%	n	%	n	%	n	%	n	%	n	
Total	17	11.8	6	4.2	16	11.1	18	12.5	26	18.1	30	20.8	31	21.5	144	100
Meningitis	6	8.1	6	8.1	10	13.5	15	20.3	10	13.5	16	21.6	11	14.9	74	100
Encephalitis	11	15.7	0	0.0	6	8.6	3	4.3	16	22.9	14	20.0	20	28.6	70	100

	M only		V only		ID only		M&V		M&ID		V&ID		M&V&ID		None		Total	
	n	%	n	%	n	%	n	%	n	%	n	%	n	%	n	%	n	%
Total	9	13.0	6	8.7	8	11.6	0	0.0	8	11.6	0	0.0	0	0.0	38	55.1	69	100
Meningitis	7	18.9	0	0.0	2	5.4	0	0.0	4	10.8	0	0.0	0	0.0	24	64.9	37	100
Encephalitis	2	6.3	6	18.8	6	18.8	0	0.0	4	12.5	0	0.0	0	0.0	14	43.8	32	100

	M not V		V not M		O		Total	
	n	%	n	%	n	%	n	%
Total	17	24.6	6	8.7	46	66.7	69	100
Meningitis	11	29.7	0	0.0	26	70.3	37	100
Encephalitis	6	18.8	6	18.8	20	62.5	32	100

n: number of studies; %: percentage total; M: microbiology; V: virology; ID: infectious diseases; N: neurology; P: paediatrics; R: research/public health; O: other.

Articles on meningitis vs encephalitis had different reported author affiliations, categorized as ‘microbiology without virology author’ (11 [29.7%] vs 6 [18.8%]) vs ‘virology without microbiology author’ (0 [0.0%] vs 6 [18.8%]) vs ‘other’ (26 [70.3%] vs 20 [62.5%]) (p=0.018). No authors affiliated with a virology department were found in studies examining the aetiology of meningitis. Additionally, a higher proportion of neurologists were involved in studies of encephalitis as compared with meningitis (15 [40.5%] vs 3 [9.4%]) (p<0.001).

## Discussion

This relatively crude analysis is consistent with an association between the study of meningitis by microbiologists and encephalitis by virologists. This probably reflects the historic prioritization of bacterial meningitis for investigation by microbiologists owing to the high untreated mortality and the need for urgent antibiotic therapy.^8^ Bacterial meningitis is regarded as more life-threatening than viral meningitis.^3^ The significance of viral meningitis, particularly the contribution of enteroviruses, is becoming better understood.^4,9^ It is increasingly recognized that vituperative pathogens such as the viral haemorrhagic fever group and arboviruses such as *Japanese encephalitis virus* may present as meningitis.^10,11^ Similarly, syphilis, leptospiral and rickettsial pathogens represent potentially treatable aetiologies of encephalitis.^12,13^

Limitations of the study include that only 17 years of publications were included and all of these were in English. The structure of the workforce in these specialities and the predominance of microbiologists over virologists worldwide is likely to have affected the results.^14–16^ Microbiologists are likely to have had training in virology and virologists training in bacteriology. The definitions of encephalitis and meningitis as given in the individual papers were used. The determination of author speciality by their reported affiliations may have led to bias: author affiliation is a crude proxy measure of speciality and some authors are likely to have had multiple specialities. For example, both microbiologists and virologists may work in infectious disease departments and physicians in both specialities may also be certified in infectious diseases. Thirty-eight (55%) studies were classified as ‘no infection input’, including those that gave hospital affiliation only rather than departmental specialty. While we focussed our analysis on infection input, this issue extends to the importance of involvement of neurologists and paediatricians.

The data questions established norms on the research evidence base for diagnosis and treatment of two important clinical CNS infection syndromes with implications for both clinical practice and research. Syndromic approaches certainly have roles, particularly in low-resource settings with poor access to laboratory diagnostics, but they have important limitations.^17–19^

These data suggest that encephalitis–virus and meningitis–bacteria associations have tended to remain compartmentalized, when in reality both clinical syndromes and aetiologies are parts of overlapping continuous spectra. Stronger multidisciplinary collaboration in the design and interpretation of CNS infection studies would avoid the pitfalls of compartmentalization.
